# MiR‐21 improves invasion and migration of drug‐resistant lung adenocarcinoma cancer cell and transformation of EMT through targeting *HBP1*

**DOI:** 10.1002/cam4.1294

**Published:** 2018-04-16

**Authors:** Chongyu Su, Xu Cheng, Yunsong Li, Yi Han, Xiaoyun Song, Daping Yu, Xiaoqing Cao, Zhidong Liu

**Affiliations:** ^1^ Department of Thoracic Surgery Beijing Chest Hospital Capital Medical University Beijing 101149 China

**Keywords:** Drug resistance, epithelial–mesenchymal transition, *HBP1*, lung adenocarcinoma, miRNA‐21

## Abstract

This study was aimed at the investigation of the effects of miR‐21 on drug resistance, invasion, migration, and epithelial–mesenchymal transition (EMT) of lung adenocarcinoma cells and the related molecular mechanisms. Cell viability of A549 cell line was measured by MTT assay. Wound healing assay and transwell assay were, respectively, employed to examine cell migration and invasion abilities. The cells were transfected with miR‐21 mimic or inhibitor using Lipofectamine 3000. The target relationship between miR‐21 and *HBP1* was confirmed by luciferase reporter gene assay. Western blot and qRT‐PCR were used to examine the expression of *HBP1* and EMT‐related molecules. Compared with A549 cells, drug resistance of A549/PTX cells and A549/DDP cells were obviously stronger. A549/PTX cells and A549/DDP cells had stronger ability of migration and invasion compared with parental A549 cells. Meanwhile, EMT of A549/PTX and A549/DDP was significantly higher than that of A549 cells. MiR‐21 promoted migration, invasion, and EMT of human lung adenocarcinoma cancer cells. Our experiment also verified the target relationship between miR‐21 and *HBP1*. MiR‐21 may affect migration and invasion ability of drug‐resistant lung adenocarcinoma cells by targeting *HBP1*, therefore modulating EMT.

## Introduction

Lung cancer is the leading cause of cancer‐related mortality worldwide. The number of deaths from lung cancer in middle urbanized areas of China in 2013 was 36,200 with mortality of 47.79/100,000 1. The proportion of lung adenocarcinoma, a major subtype of non–small‐cell lung cancer (NSCLC) 2, has raised about 40% among lung cancer since 2013 3. Great progress has been made in early diagnosis, surgical techniques, and targeted therapy, whereas the prognosis for patients with NSCLC remains poor with 15% of 5‐year survival rate 4. The classification of lung adenocarcinoma has been revised to incorporate important new elements by the World Health Organization (WHO), which has prognostic significance and might help predict therapeutic approaches 5. The poor outcome is mainly accounted for by chemoresistance, an inevitable occurrence in lung adenocarcinoma 6. Li et al. reported that cisplatin (DDP) or paclitaxel (PTX) drug exposure gave rise to lung adenocarcinoma cells with aggressiveness and metastatic potential 7. Thus, it is of the utmost importance for deeper understanding of novel molecular mechanisms to overcome drug resistance in lung adenocarcinoma cells.

A category of small endogenous noncoding RNAs, approximately 19–25 nucleotides in length, which regulate the expression of their target genes at the post‐transcriptional level, are called microRNAs (miRNAs). MiRNAs play an important part in various biological processes such as cell proliferation, migration, differentiation, and apoptosis 8, 9. Beyond the involvement in physiological processes, accumulating evidence strongly suggests that the dysregulation of miRNAs may bring about the initiation and progression of cancer, which makes them valuable cancer biomarkers 10. In vivo and in vitro studies revealed that miR‐21 could serve as a diagnostic or prognostic marker for human malignancies such as tongue squamous cell carcinoma (SCC), NSCLC, chronic lymphocytic leukemia, and pancreatic cancer 11, 12, 13, 14, 15. Besides, miR‐21 overexpression is associated with EGFR–TKI‐resistant human lung adenocarcinoma cell line 16. However, the role of miR‐21 and how it works in lung adenocarcinoma remain unclear.

HMG box transcription factor 1 (*HBP1*) is a member of the high‐mobility group protein family, which is a transcriptional repressor and contains 513 amino acid residues of polypeptide 17, 18. By regulating transcription of various genes, *HBP1* has been found to participate in multiple cell progressions including cell cycle inhibition, terminal differentiation, senescence induction, and tumor suppression in a variety of tissues and cell types 19, 20, 21. *HBP1* pathway could also be modulated by other factors, therefore influencing cancer development. For instance, *HBP1‐p53‐Srebp1c* pathway could be regulated by miR‐21 to affect hepatocellular carcinoma progression 22. Nevertheless, no study has thoroughly elaborated the role of *HBP1* during lung adenocarcinoma development.

Epithelial–mesenchymal transition (EMT) is the conversion of epithelial cells to mesenchymal cells, in which cells undergo physiological or pathological changes including the loss of cell polarity and cell–cell adhesion as well as the acquisition of migratory and invasive properties 23. Thus, EMT has recently been recognized to be highly responsible for carcinoma progression in several types of cancer, including non–small‐cell lung cancer (NSCLC) 24. On the other hand, the EMT‐induced stemness endows cancer cells with the ability to overexpress chemoresistance‐related genes, leading to multiple drug resistance in cancer treatment. Both the development of drug resistance and the occurrence of EMT are negative effects induced by chemotherapy. It has been reported that EMT is associated with reduction of drug sensitivity and acquisition of resistance in lung adenocarcinoma 25. Taken together, several studies have demonstrated that EMT not only enhances the metastatic potentials of cancer, but also participates in the development of chemoresistance 26.

Our study herein was conducted to evaluate the biological roles of miR‐21/*HBP1* in lung adenocarcinoma growth, migration, and invasion, and to identify *HBP1* as a target of miR‐21. We also tried to find the association of EMT and the above two factors miR‐21/*HBP1*. The regulatory mechanism between *HBPI* and miR‐21 found in our study might be a therapeutic target for patients with NSCLC, which would further control the recurrent and improve the prognosis of lung adenocarcinoma.

## Materials and Methods

### Cell lines and cell culture

Human lung cancer cell line A549 was obtained from BeNa Culture Collection (BNCC; Beijing, China). The cell line was confirmed by short tandem repeat profiling and tested for mycoplasma contamination. Paclitaxel (PTX) was purchased from Beijing Pharmaceutical (Beijing, China) and cisplatin (DDP) was procured from Qilu Pharmaceutical (Jinan, Shandong, China). Cells were cultured in RPMI 1640 (Gibco, Gaithersburg, MD) containing 10% FBS, 100 U/mL penicillin, and 100 U/mL streptomycin, subcultured every 3–4 days, and incubated at 37°C in a humidified environment. A549 cells were continuously cultured with gradient concentration of PTX and DDP for more than 12 months until the cells showed the drug resistance against 200 *μ*g/mL of PTX or 1000 *μ*g/mL of DDP. Thereafter, PTX‐resistant A549 (A549/PTX) cell line and DDP‐resistant A549 (A549/DDP) cell line were screened out and prepared for subsequent experiments.

### Cell transfection

MiR‐21 mimic, miR‐21 inhibitor, control mimic, and control inhibitor were synthesized by GenePharma (Shanghai, China). A549 cells were placed on six‐well plates (2 × 10^5^ cells/well) and incubated for 24 h until 50–80% confluence. The sequence of miR‐21 mimic was UAGCUUAUCAGACUGAUGUUGA, and the sequence of miR‐21 inhibitor was UCAACAUCAGUCUGAUAAGCUA. Lipofectamine 3000 (Invitrogen, Gaithersburg, MD) was applied to cell transfection following the manufacturer's instructions. The grouping of cells was as follows: blank group (nontransfection), mimic NC group (transfected with miR‐21 control mimic), inhibitor NC group (transfected with miR‐21 control inhibitor), miR‐21 mimic group (transfected with miR‐21 mimic), and miR‐21 inhibitor group (transfected with miR‐21 inhibitor). Analyses were conducted 24–48 h later after transfection.

### Cell proliferation assay

After transfected with miR‐21 mimic or miR‐21 inhibitor, A549 cells were first harvested, seeded into 96‐well plates (7 × 10^3^ cells/well), and incubated overnight. Next, the cells were treated with different concentrations of PTX or DDP and then 20 *μ*L of MTT (Sigma‐Aldrich, St. Louis, MO) was added into each well and incubated for 4 h. Afterward, the supernatant was discarded and 150 *μ*L of dimethyl sulfoxide (DMSO) was added into each well. After the crystals were dissolved, the cells were observed at 24 h, 48 h, and 72 h under a microscope, and half maximal inhibitory concentration (IC_50_) and resistance index (RI) were calculated. The optical density (OD) was measured at 490 nm.

### Wound healing assay

A549 cell, A549/PTX cells, and A549/DDP cells were put on the six‐well plate 24 h post‐transfection and then cultured for 24 h until 90% confluence. A scratch was created using a sterile 200 mL micropipette tip. The cells were subsequently rinsed twice with serum‐free medium to remove cell debris. Afterward, the cells continued to be incubated in an incubator at room temperature. The scratch healing areas of cells were observed among different transfection groups and during different time points. Finally, three randomly selected fields were photographed by an inverted microscope (BD Biosciences, San Jose, CA).

### Transwell assay

A 24‐pore transwell chamber (Corning Inc., NY, USA) with polycarbonate membrane filter covered with the gelatin package was used to measure the migration ability of A549 cells, A549/PTX cells, and A549/DDP cells. After diluted with 100 *μ*L serum‐free medium, Matrigel (BD Biosciences) was used to cover the bottom membranes of transwell chamber. After that, 2.5 × 10^4^ cells were inoculated onto the upper chamber. The serum and growth factors such as chemokines were added in the lower chamber and incubated overnight. The noninvading cells on the transwell chamber were wiped off with cotton swab after incubation, while the migratory or invasive cells were fixed with 4% paraformaldehyde and then stained with Giemsa stain (Keygentec, Jiangsu, China). The migratory or invasive cells were counted through a microscope (×400).

### RNA extraction and reverse transcription quantitative polymerase chain reaction

For the reverse transcription quantitative polymerase chain reaction (qRT‐PCR) analysis of miRNAs, total RNA including miRNA from the tissues and cultured cells was extracted through mirVana^™^ miRNA isolation kit (AM1560; Ambion, Austin, TX) following the producer's guideline. After being reverse transcribed by miScript Reverse Transcription II Kit (Qiagen), specialized qRT‐PCR kits (Qiagen) for miRNA were utilized to measure expression levels of miR‐21 mRNA. U6 small nuclear RNA was used as an internal control. For the qRT‐PCR analysis of mRNAs, total RNA was isolated from harvested cells using Trizol (Invitrogen) followed by further purification with RNeasy mini kit and RNase‐free DNase Set (Qiagen, Shanghai, China) in accordance with the instructions. To determine the mRNA levels in A549, A549/PTX, and A549/DDP cells, total RNAs were treated with DNase I (AM1906; Ambion) to eliminate genomic DNA contamination, and then reversely transcribed using Advantage RT‐4PCR kit (Clontech, Mountain View, CA). The results were normalized to the expression of GAPDH. Primers designed and used in qRT‐PCR were displayed in Table 1. Results were calculated using 2^−ΔΔCt^ method.

**Table 1:** The primer sequences of qRT‐PCR products

Gene	Primer sequence (5′–3′)
*miR‐21*	F: AGAAATGCCTGGGTTTTTTTGGTTR: TTGGGAATGCTTTTCAAAGAAGGT
*U6*	F: CTCGCTTCGGCAGCACACAR: AACGCTTCACGAATTTGCGT
*PBR1*	F: TGAAGGCTGTGATAATGAGGAAGATR: CATAGAAAGGGTGGTCCAGCTTA
*E‐cadherin*	F: CATTTCCCAACTCCTCTCCTGGCR: ATGGGCCTTTTTCATTTTCTGGG
*β*‐*catenin*	F: CACAAGCAGAGTGCTGAAGGTGR: GATTCCTGAGAGTCCAAAGACAG
*Vimentin*	F: AGATGGCCCTTGACATTGAGR: TGGAAGAGGCAGAGAAATTC
*MMP*‐2	F: GATAACCTGGATGCCGTCGTGR: CTTCACGCTCTTCAGACTTTGGTTC
*MMP*‐9	F: CGGAGTGAGTTGAACCAGR: GTCCCAGTGGGGATTTAC
*Snail*	F: CCAGCTCTCTGAGGCCAAGGATCR: TGGCTTCGGATGTGCATCTTGAG
*Slug*	F: CCCTGAAGATGCATATTCGGACR: CTTCTCCCCCGTTGTAGTTCTA
*Twist*	F: TGCGGAAGATCATCCCCAR: TCCATCCTCCAGACCGAGAA
*ZEB1*	F: GCACAACCAAGTGCAGAAGAR: GCCTGGTTCAGGAGAAGATG
*GAPDH*	F: AAGGTGAAGGTCGGAGTCAACR: CTTGATTTTGGAGGGATCTCG

F, forward primer, R, reverse primer.

### Differential expression analysis

Total RNA for each of the three kinds of cultured cells (A549, A549/PTX, and A549/DDP cells) was divided into two parts, one for miRNA sequencing and the other for mRNA sequencing using the Illumina HiSeq2000 sequencing system at the BGI in Shenzhen, China. The Illumina HiSeq2000 system was a high‐throughput sequencing technology based on massive parallel sequencing of millions of fragments using proprietary reversible terminator‐based sequencing chemistry, the same sequencing principle used in the Illumina Genome Analyzer II system. The RNA sequencing process was performed according to the manufacturer's protocols as well as standardized protocols developed by the core laboratory at the BGI. Differentially expressed mRNAs and miRNAs were screened out from the expression data of the above three kinds of cell lines, with each kind including five groups of cells. Fold‐change (FC) value >2 and *P *< 0.05 were the screening conditions to identify the genes and miRNAs with differential expression, using the R project for statistical computing. On the other hand, data for prognostic and survival analysis of patients were obtained from The Cancer Genome Atlas (TCGA) database (https://tcga-data.nci.nih.gov/tcga/).

### Western blot

Cells were placed on the six‐well culture plate (4 × 10^5^ cells/hole) (Corning Inc.) and lysed using RIPA buffer (Sigma). BCA (Beyotime, Jiangsu, China) protein kit was used to measure protein concentration. After that, proteins were detached using SDS‐PAGE (Bio‐Rad, Hercules, CA) and then transferred onto polyvinylidene fluoride (PVDF) membranes (Millipore, Bedford). After soaked in 5% skim milk for 1 h in Tris‐buffered saline–Tween 20 (TBST), the membranes were incubated using primary antibodies anti‐E‐cadherin (ab133597, 1:1000, Abcam, CA, MA), anti‐*β*‐catenin (ab6302, 1:4000, Abcam), anti‐MMP‐2 (ab175937, 1:1000, Abcam), anti‐MMP‐9 (ab373734, 1:1000, Abcam), anti‐Snail (ab82846, 1:500, Abcam), anti‐Slug (ab183760, 1:1000, Abcam), anti‐Vimentin (ab137321, 1:1000, Abcam), anti‐Twist (ab49254, 1:1000, Abcam), anti‐ZEB1 (ab203829, 1:1000, Abcam), and anti‐*β*‐actin (ab8227, 1:3000, Abcam) at 4°C for 24 h. After washed three times using TBST, the membranes were incubated with secondary antibody HRP‐labeled goat anti‐rabbit IgG H&L (1:2000) antibody, which was added to incubate the membranes for 1 h. Then, it was washed for 10 min in TBST. Protein bands were visualized by ECL Plus Western Blotting Substrate (Thermo Fisher Scientific, MA). *β*‐Actin was deemed an internal control.

### Dual‐luciferase reporter gene assay

PCR was used to amplify *HBP1* wild‐type and mutated 3′ UTR binding sites of miR‐21‐3p. *HBP1* 3′ UTR wild type (*HBP1* 3′ UTR wt) and mutated (*HBP1* 3′ UTR mut) were cloned into pGL3‐*HBP1* vector. When cell growth reached 80% confluence, 1 × 10^6^ cells were cotransfected with 50 pmol miR‐21‐3p mimic or mimic‐NC and 1 *μ*g pGL3‐*HBP1*‐wt or pGL3‐*HBP1*‐mut plasmid using Lipofectamine 2000 reagent. After incubation for 36 h post‐transfection, dual‐luciferase reporter assay system (Promega, Southampton, UK) was used to evaluate luciferase activity. Results were unified as Renilla luciferin enzyme activity.

### Statistical analysis

SPSS version 20.0 statistical software (SPSS, Chicago, IL) was applied for statistical analyses. Experimental data were calculated as mean ± SD. Statistical differences between groups were compared by one‐way ANOVA and statistical differences among groups were compared by Student's *t*‐test. *P* < 0.05 denoted a statistical significance.

## Results

### Establishment of A549 drug‐resistant cell lines

**Figure 1:**
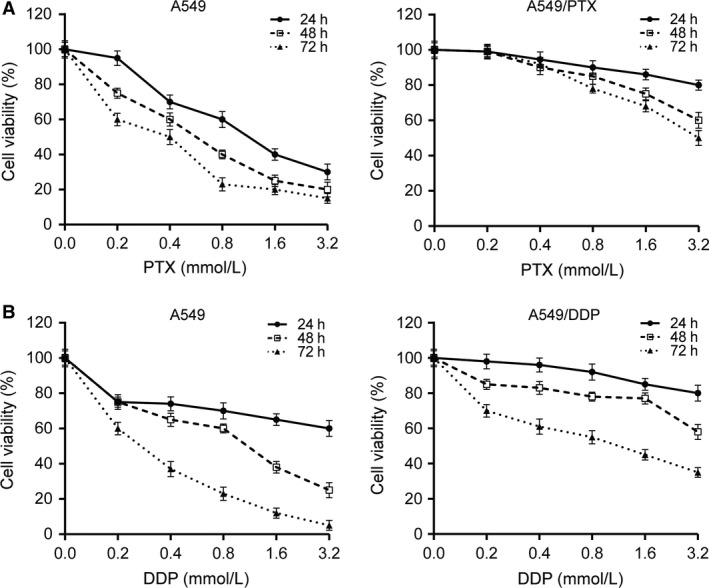
The cell viability of A549 cells treated with different concentrations of cisplatin (DDP) or paclitaxel (PTX). The result of MTT assay showed that the IC_50_ of (A) A549/PTX and (B) A549/DDP was obviously stronger than that of A549 parental cells at 24 h, 48 h, and 72 h, respectively.

To measure the drug resistance of A549 cell lines, we cultured cells with PTX or DDP in different concentrations (0.0, 0.2, 0.4, 0.8, 1.6, and 3.2 mmol/L) for 24 h, 48 h, and 72 h. The result of MTT assay showed that the drug resistance of A549/PTX cells and A549/DDP cells were obviously stronger than that of A549 cells (Fig. 1), which indicated that the drug‐resistant cell lines were established successfully. Compared with A549 parental cells, higher IC_50_ was observed in A549/PTX cells and A549/DDP cells at 24 h, 48 h, and 72 h, respectively (*P *< 0.01). At the same time, A549/PTX cells and A549/DDP cells had higher RI (Tables 2 and 3). PTX or DDP drug‐resistant cell line established successfully was isolated for the preparation for follow‐up experiments.

**Table 2:** IC_50_ and the drug resistance index of A549 and A549/PTX cells treated with PTX for 24 h, 48 h, and 72 h

Time (h)	IC_50_ (*μ*mol/L)			RI
	A549	A549/PTX	*P*	
24	1.22 ± 0.46	7.3 ± 0.44	<0.01	6 ± 0.72
48	0.52 ± 0.08	3.22 ± 0.26	<0.01	6.2 ± 0.48
72	0.25 ± 0.17	2.55 ± 0.31	<0.01	10.2 ± 0.63

IC_50_, 50% inhibition concentration; RI, drug resistance index; PTX, paclitaxel. *P *< 0.05, compared with A549 group.

**Table 3:** IC_50_ and the drug resistance index of A549 and A549/DDP cells treated with DDP for 24 h, 48 h, and 72 h

Time (h)	IC_50_ (*μ*mol/L)			RI
	A549	A549/DDP	*P*	
24	75.83 ± 0.53	250.24 ± 0.94	<0.01	3.3 ± 0.74
48	17.62 ± 0.12	66.96 ± 0.59	<0.01	3.8 ± 0.42
72	5.84 ± 0.32	54.90 ± 0.63	<0.01	9.4 ± 0.83

IC50, 50% inhibition concentration; RI, drug resistance index; DDP, cisplatin. *P *< 0.05, compared with A549 group.

### Migration, invasion, and EMT of A549/PTX and A549/DDP cell lines

**Figure 2:**
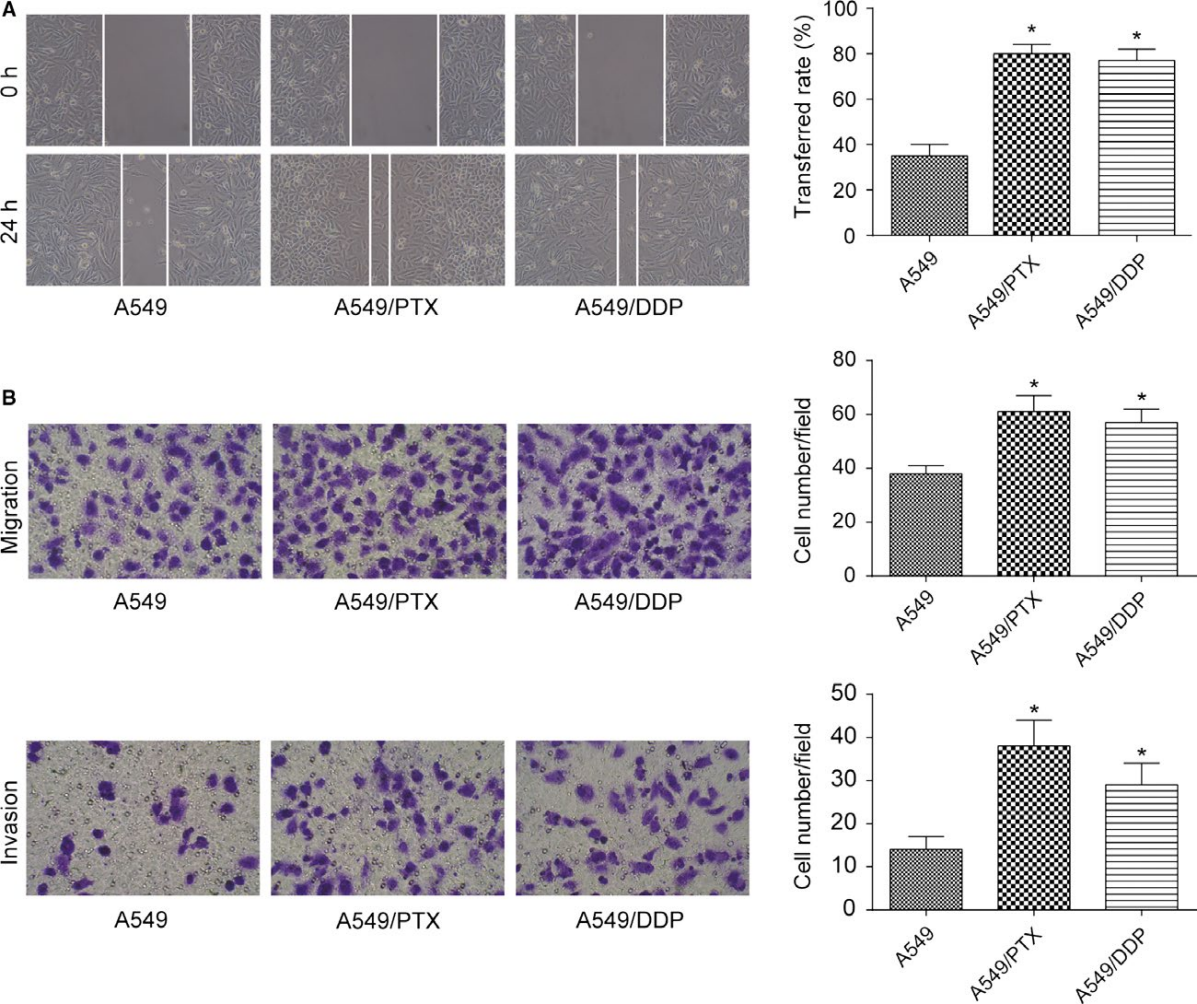
A549/PTX and A549/DDP cells showed stronger ability of migration and invasion. (A) Wound healing assay determined that A549/PTX and A549/DDP cells had longer migration distance compared with parental A549 cells (×100). (B) After incubating 24 h with transwell chambers, the number of migrated and invaded A549/PTX and A549/DDP cells were markedly increased compared with parental A549 cells (×200). *P < 0.05, compared with A549 group.

**Figure 3:**
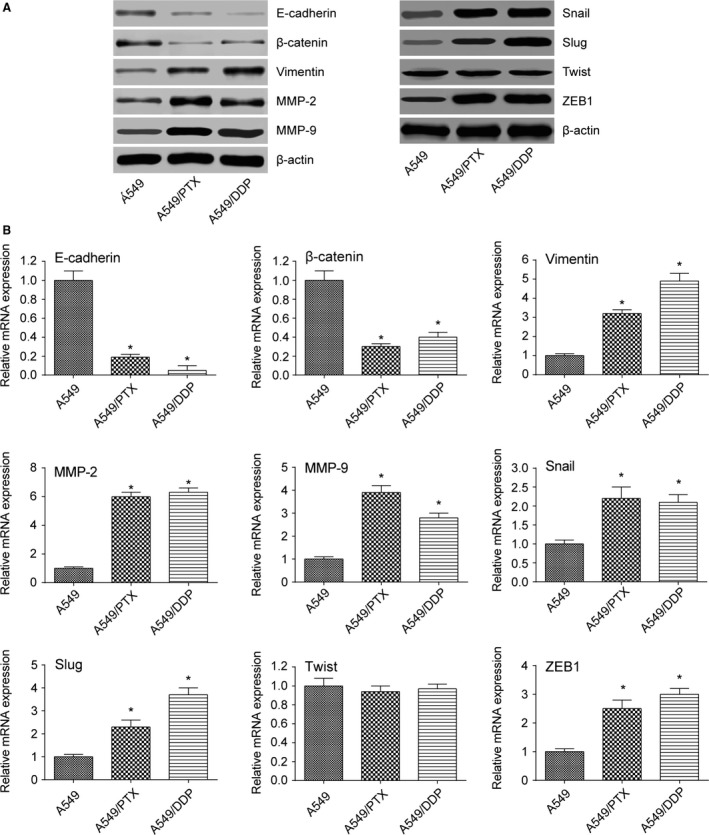
The expression level of EMT‐related markers in A549/DDP and A549/PTX cells. (A) Western blot showed that the expression of E‐cadherin and *β*‐catenin in A549/PTX and A549/DDP cells was dramatically downregulated, while the expression of EMT‐related markers except Twist was significantly upregulated. (B) qRT‐PCR showed that the expression of E‐cadherin and *β*‐catenin in A549/PTX and A549/DDP cells was dramatically downregulated, while the expression of EMT‐related markers except Twist was significantly upregulated. *P < 0.05, compared with A549 group.

Compared with parental A549 cells, stronger ability of migration and invasion was observed in A549/PTX and A549/DDP cells by the detection of wound healing assay and transwell assay (all *P *< 0.05, Fig. 2A and B). The expression of EMT markers was further measured by the method of western blot and RT‐PCR. The results showed that compared with A549 cell lines, the expression of epithelial adhesion molecules E‐cadherin and *β*‐catenin in A549/PTX and A549/DDP cells was dramatically downregulated, while the expression of mesenchymal markers except for Twist (Vimentin, MMP‐2, MMP‐9, Snail, Slug, and ZEB1) was significantly upregulated (all *P *< 0.05, Fig. 3A and B).

### MiR‐21 promoted migration and invasion of lung adenocarcinoma cancer cells

**Figure 4:**
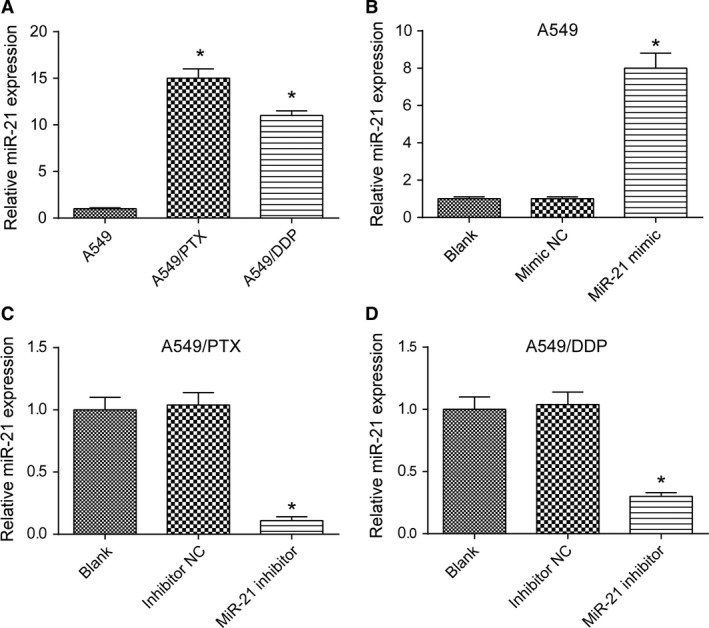
The expression level of miR‐21 in A549, A549/DDP, and A549/PTX cells. (A) qRT‐PCR showed that the expression level of miR‐21 in A549/DDP and A549/PTX cells was significantly higher than that in A549 cells. *P < 0.05, compared with A549 group. (B) The expression of miR‐21 in A549 cells was raised with the transfection of miR‐21 mimic. *P < 0.05, compared with mimic NC group. (C, D) After transfected with miR‐21 inhibitor, the expression of miR‐21 in A549/PTX and A549/DDP cells was notably decreased. *P < 0.05, compared with inhibitor NC group.

**Figure 5:**
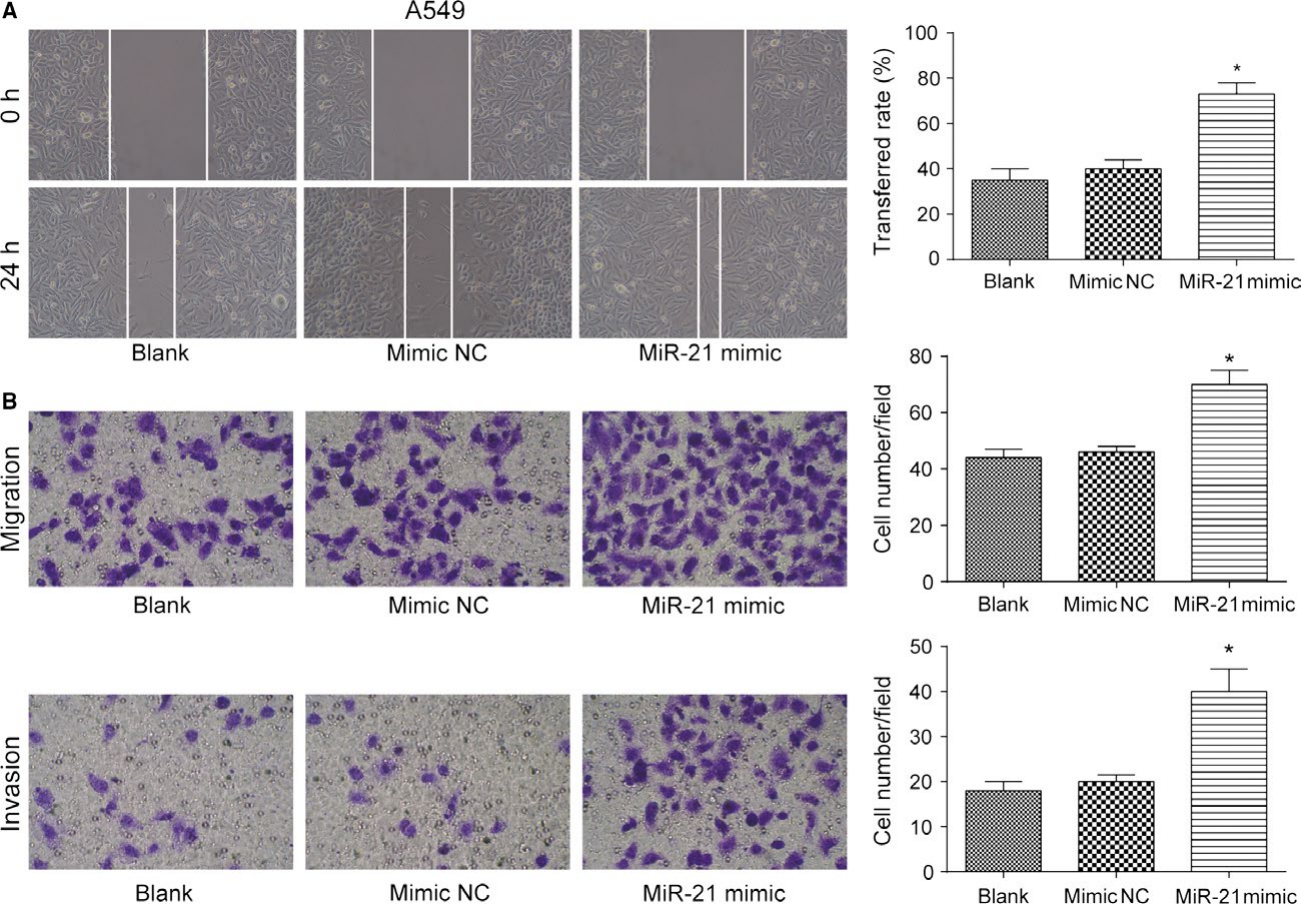
The effect of miR‐21 on the ability of invasion and migration of A549 cells. (A) The migration distance of A549 cells in miR‐21 mimic group was more than that in mimic NC group (×100). (B) The number of migrated and invaded A549 cells in miR‐21 mimic group was more than that in mimic NC group (×200). *P < 0.05, compared with mimic NC group.

**Figure 6:**
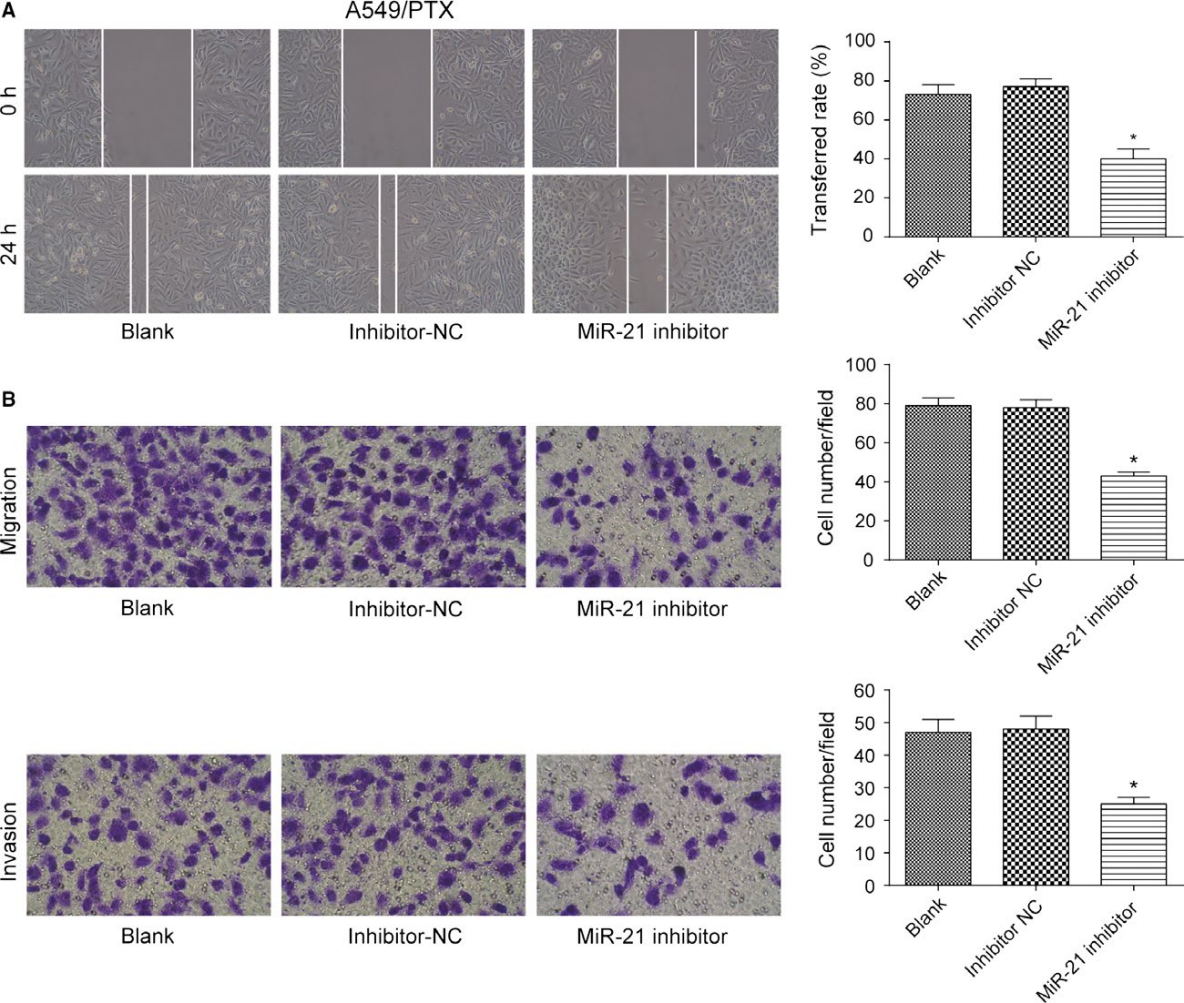
The effect of miR‐21 on the ability of invasion and migration of A549/PTX cells. (A) The migration distance of A549/PTX cells in miR‐21 inhibitor group was less than that in inhibitor NC group (×100). (B) The number of migrated and invaded A549/PTX cells in miR‐21 inhibitor group was observably less than that in inhibitor NC group (×200). *P < 0.05, compared with inhibitor NC group.

**Figure 7:**
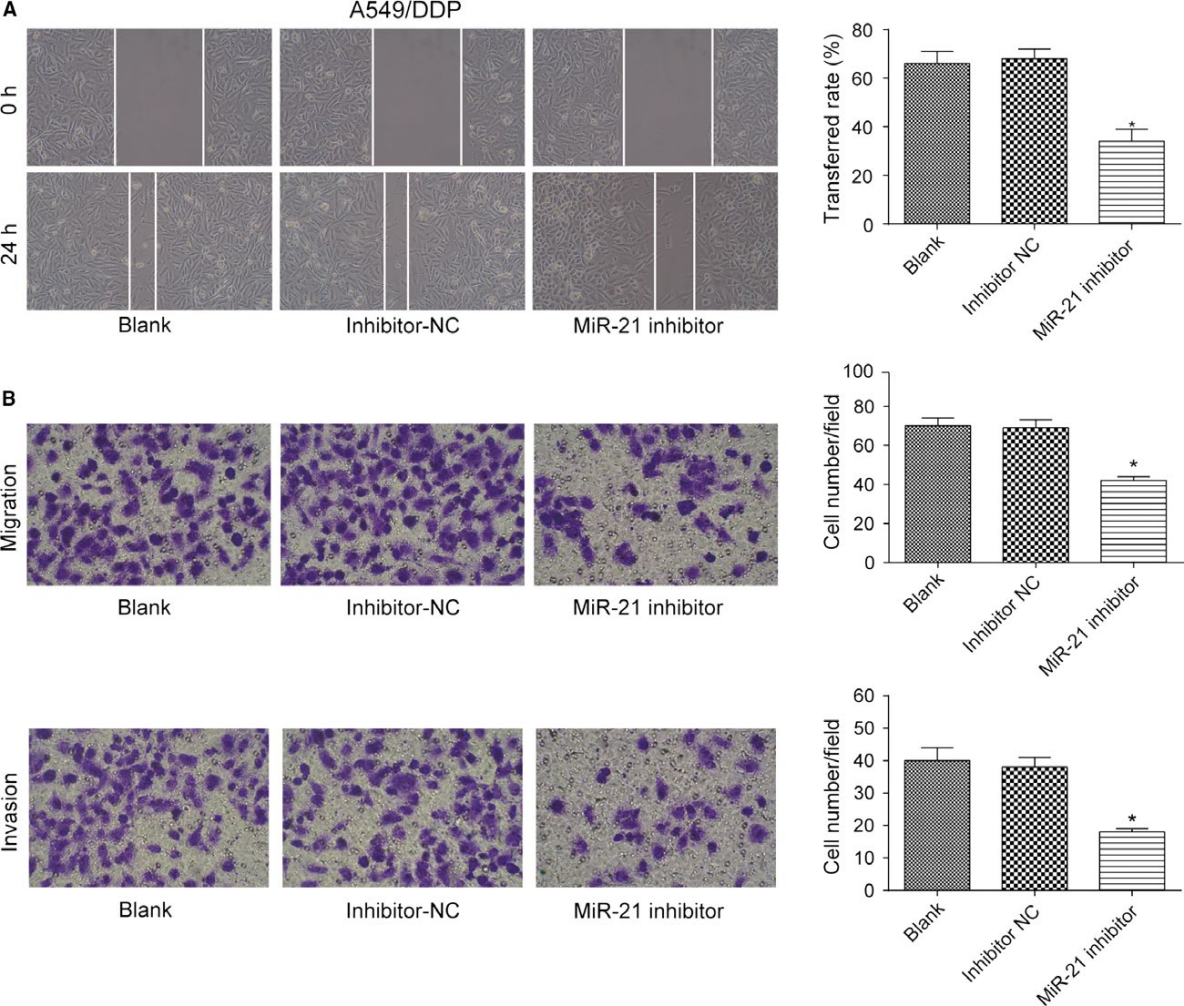
The effect of miR‐21 on the ability of invasion and migration of A549/DDP cells. (A) The migration distance of A549/DDP cells in miR‐21 inhibitor group was less than that in inhibitor NC group (×100). (B) The number of migrated and invaded A549/DDP cells in miR‐21 inhibitor group was observably less than that in inhibitor NC group (×200). *P < 0.05, compared with inhibitor NC group.

In five cases of A549, A549/PTX, and A549/DDP cells, expression of 515 miRNAs was upregulated, while expression of 361 miRNAs was downregulated (Fig. S1A). The expression of miR‐21 in drug‐resistant A549/PTX and A549/DDP cells was significantly increased by 1.997 times (*P *= 3.04E‐18). The heat map showed the miRNAs with the most significant differential expression (Fig. S1B). TCGA analysis displayed that patients with higher level of miR‐21 had poorer prognosis and shorter survival time (Fig. S1C). qRT‐PCR showed that the expression of miR‐21 in A549/PTX cells and A549/DDP cells was obviously upregulated compared with A549 cells (both *P *< 0.05, Fig. 4A). Our experiment results showed that miR‐21 mimic dramatically enhanced the expression of miR‐21 in A549 cells (*P *< 0.05, Fig. 4B), and miR‐21 inhibitor effectively suppressed the expression of miR‐21 in A549/PTX cells and A549/DDP cells (both *P*s* *< 0.05, Fig. 4C and D). MiR‐21 mimic improved the ability of invasion and migration of A549 cells (all *P*s* *< 0.05, Fig. 5A and B), and miR‐21 inhibitor weakened the ability of invasion and migration of A549/PTX cells (all *P*s* *< 0.05, Fig. 6A and B) and A549/DDP cells (all *P*s* *< 0.05, Fig. 7A and B). These results showed that miR‐21 was the upstream regulation factor of invasion and migration of lung adenocarcinoma cancer cells, drug sensitive or drug resistant.

### MiR‐21 promoted the viability and EMT of lung adenocarcinoma cancer cells

**Figure 8:**
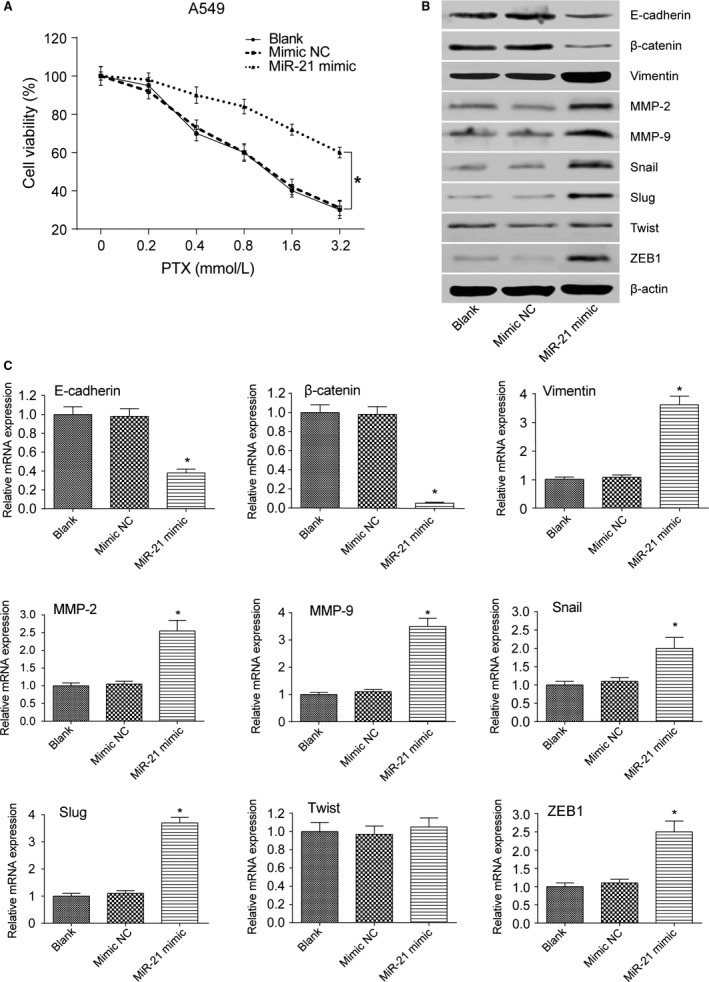
The effect of miR‐21 on EMT of A549 cells treated with PTX. (A) MTT assay showed that A549 cells transfected with miR‐21 mimic increased cell viability after treated with PTX. (B) Western blot illustrated that A549 cells in the miR‐21 mimic group had decreased expression level of E‐cadherin and *β*‐catenin and increased expression level of EMT‐related markers except Twist. (C) qRT‐PCR verified that A549 cells in the miR‐21 mimic group had decreased expression level of E‐cadherin and *β*‐catenin and increased expression level of EMT‐related markers except Twist. *P < 0.05, compared with mimic NC group.

**Figure 9:**
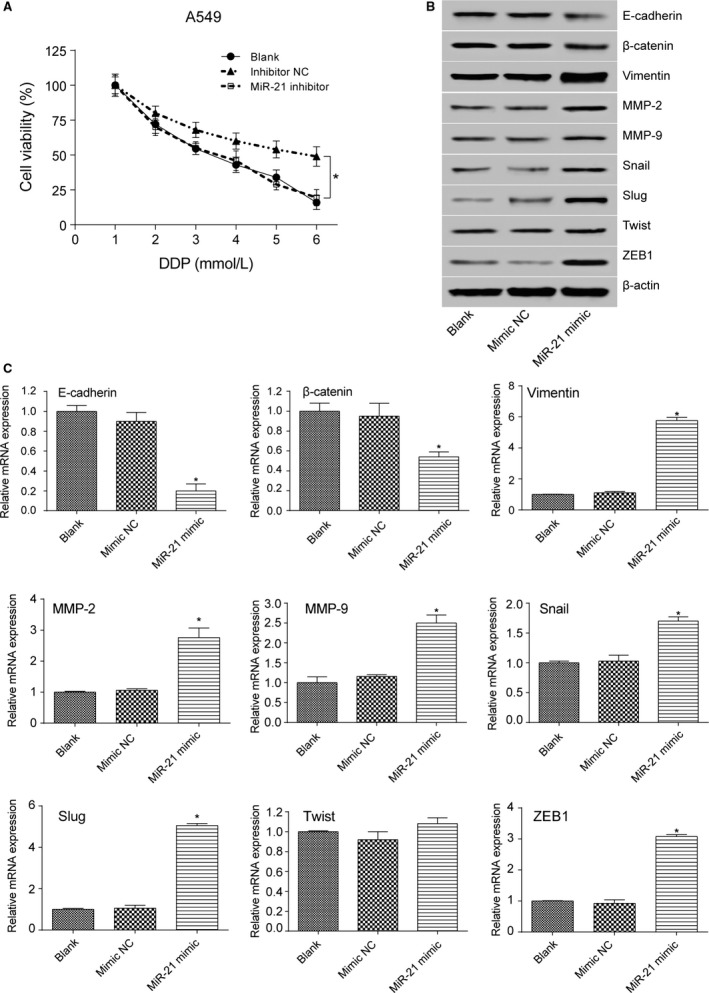
The effect of miR‐21 on EMT of A549 cells treated with DDP. (A) MTT assay showed that A549 cells transfected with miR‐21 mimic increased cell viability after treated with DDP. (B) Western blot illustrated that A549 cells in the miR‐21 mimic group had decreased expression level of E‐cadherin and *β*‐catenin and increased expression level of EMT‐related markers except Twist. (C) qRT‐PCR verified that A549 cells in the miR‐21 mimic group had decreased expression level of E‐cadherin and *β*‐catenin and increased expression level of EMT‐related markers except Twist. *P < 0.05, compared with mimic NC group.

**Figure 10:**
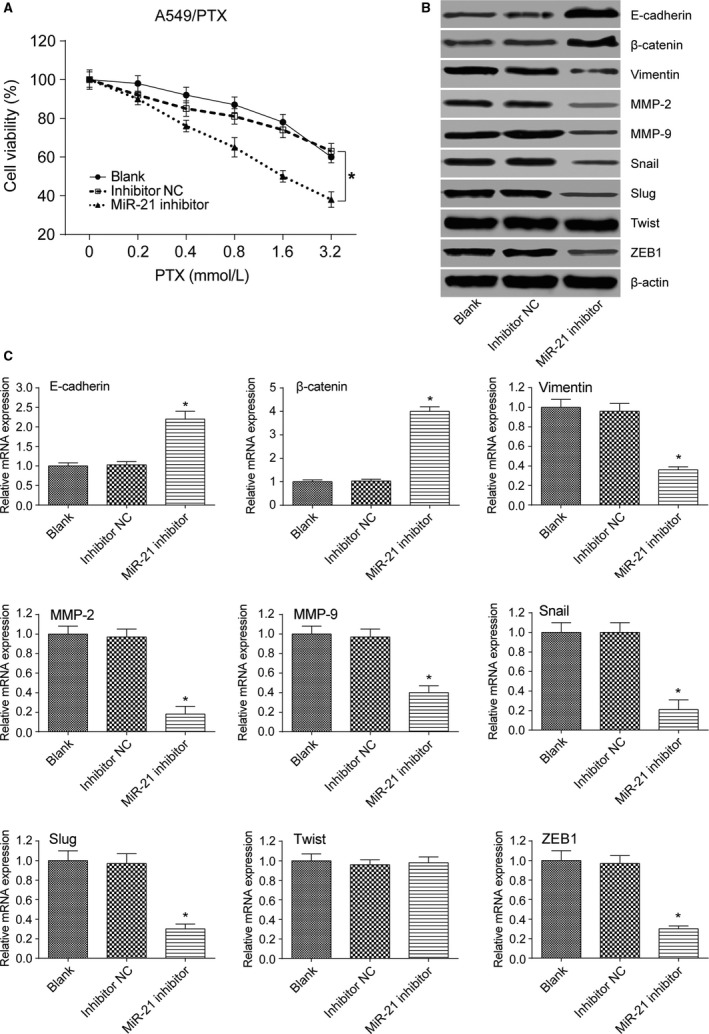
The effect of miR‐21 inhibitor on EMT of A549/PTX cells. (A) MTT assay indicated that the cell viability of A549/PTX cells transfected with miR‐21 inhibitor was decreased. (B) According to the results of western blot, A549/PTX cells transfected with miR‐21 inhibitor increased the expression level of E‐cadherin and *β*‐catenin and decreased the expression level of EMT‐related markers except Twist. (C) qRT‐PCR verified that A549/PTX cells transfected with miR‐21 inhibitor increased the expression level of E‐cadherin and *β*‐catenin and decreased the expression level of EMT‐related markers except Twist. *P < 0.05, compared with inhibitor NC group.

**Figure 11:**
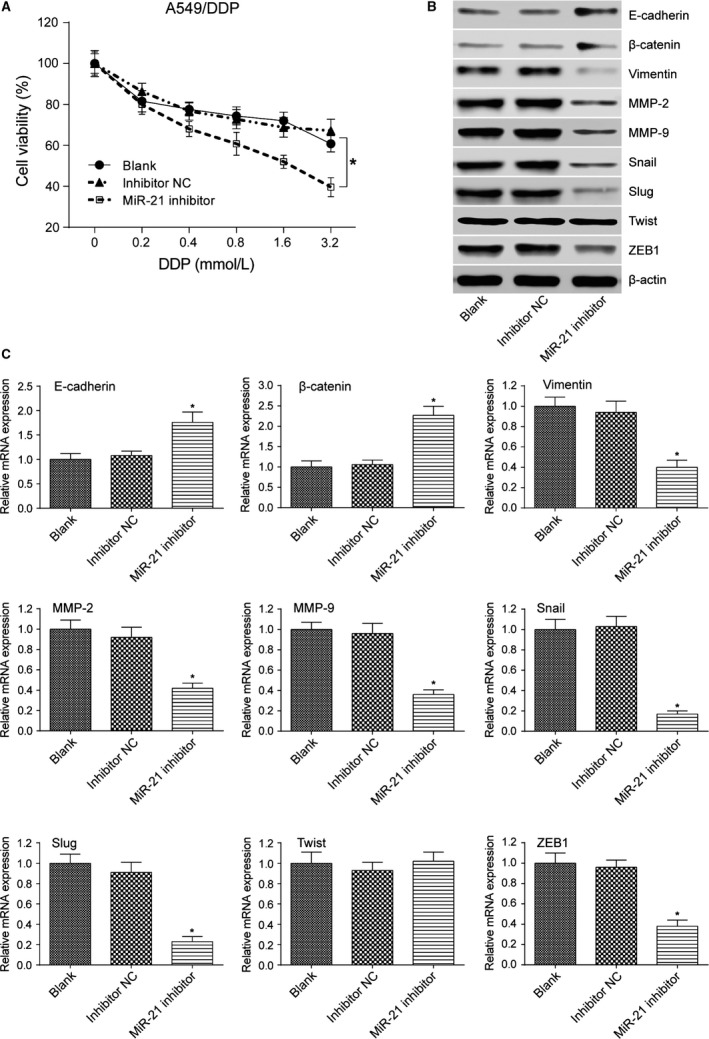
The effect of miR‐21 inhibitor on EMT of A549/DDP cells. (A) MTT assay indicated that the cell viability of A549/DDP cells transfected with miR‐21 inhibitor was decreased. (B) According to the results of western blot, A549/DDP cells transfected with miR‐21 inhibitor increased the expression level of E‐cadherin and *β*‐catenin and decreased the expression level of EMT‐related markers except Twist. (C) qRT‐PCR verified that A549/DDP cells transfected with miR‐21 inhibitor increased the expression level of E‐cadherin and *β*‐catenin and decreased the expression level of EMT‐related markers except Twist. *P < 0.05, compared with inhibitor NC group.

MTT assay result showed that A549 cells transfected with miR‐21 mimic had increased cell viability after treated with stepwise increasing concentrations of PTX (*P *< 0.05 at 0.4, 0.8, 1.6, and 3.2 mmol/L, Fig. 8A). Besides, miR‐21 mimic transfection decreased the expression level of E‐cadherin and *β*‐catenin (both *P*s* *< 0.05) and increased the expression level of mesenchymal marker molecules vimentin, MMP‐2, MMP‐9, Snail, ZEB1, and Slug (all *P*s* *< 0.05, Fig. 8B and C). MTT assay result also showed that A549 cells transfected with miR‐21 mimic had increased cell viability after treated with stepwise increasing concentrations of DDP (*P *< 0.05 at 0.4, 0.8, 1.6, and 3.2 mmol/L, Fig. 9A). Similarly, miR‐21 mimic transfection decreased the expression level of E‐cadherin and *β*‐catenin (both *P*s* *< 0.05) and increased the expression level of mesenchymal marker molecules vimentin, MMP‐2, MMP‐9, Snail, ZEB1, and Slug (all *P*s* *< 0.05, Fig. 9B and C). A549/PTX cells transfected with miR‐21 inhibitor showed decreased cell viability after treated with stepwise increasing concentrations of PTX (*P *< 0.05 at 0.4, 0.8, 1.6, and 3.2 mmol/L, Fig. 10A). MiR‐21 inhibitor transfection increased the expression level of E‐cadherin and *β*‐catenin (both *P*s* *< 0.05) and decreased the expression level of mesenchymal marker molecules vimentin, MMP‐2, MMP‐9, Snail, ZEB1, and Slug (all *P*s* *< 0.05, Fig. 10B and C). Meanwhile, MTT assay showed that A549/DDP cells transfected with miR‐21 inhibitor had decreased cell viability after treated with stepwise increasing concentrations of DDP (*P *< 0.05 at 0.4, 0.8, 1.6, and 3.2 mmol/L, Fig. 11A). MiR‐21 inhibitor transfection increased the expression level of E‐cadherin and *β*‐catenin (both *P*s* *< 0.05) and decreased the expression level of mesenchymal marker molecules vimentin, MMP‐2, MMP‐9, Snail, ZEB1, and Slug (all *P*s* *< 0.05, Fig. 11B and C). These results reflected that the overexpression of miR‐21 promoted the formation of EMT phenotype in parental A549 cells, while the inhibition of miR‐21 reversed the EMT phenotype in A549/PTX cells or A549/DDP cells.

### MiR‐21 reduced the expression level of *HBP1* by targeting *HBP1* 3′ UTR directly

**Figure 12:**
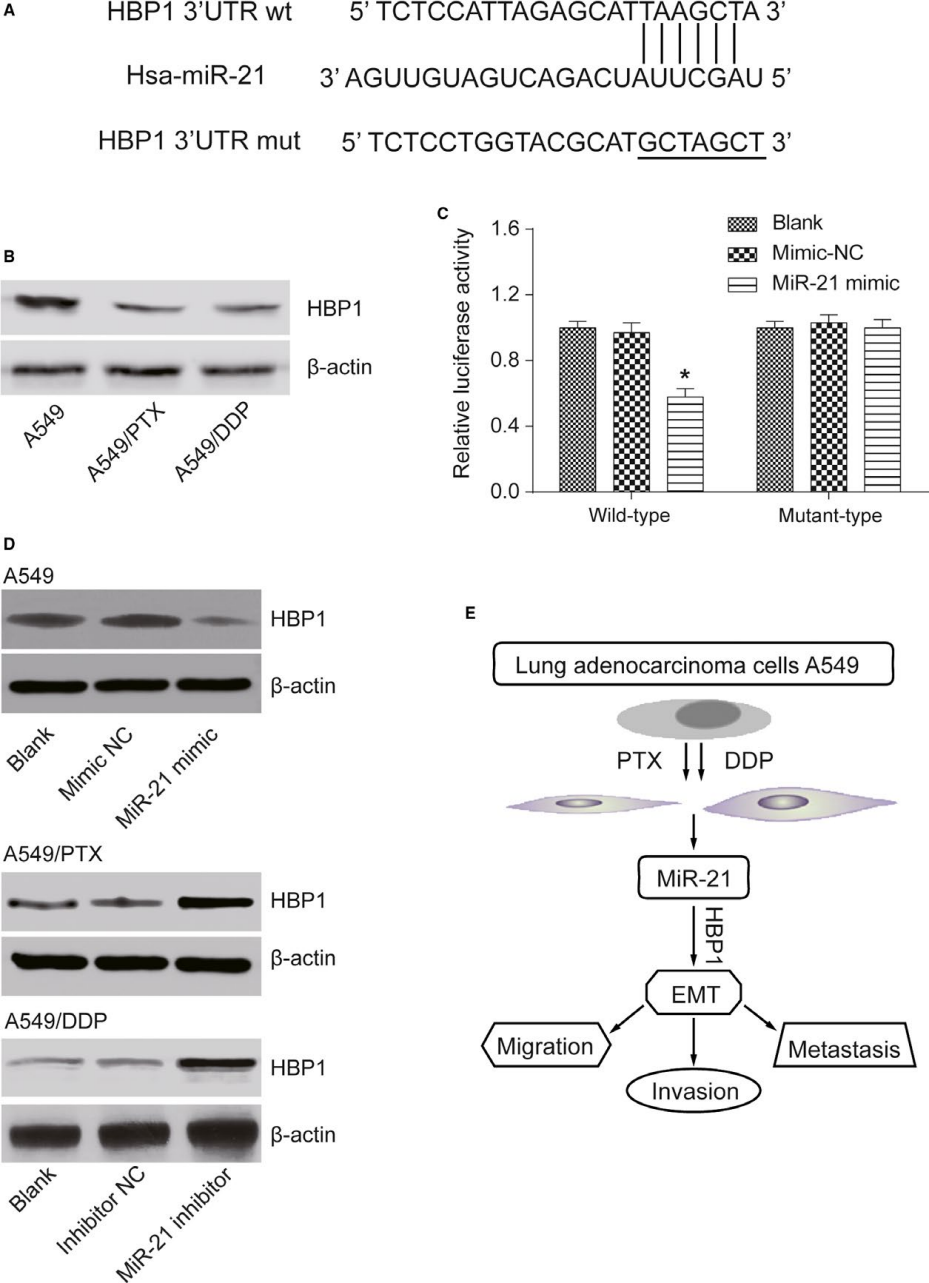
MiR‐21 directly targeted *HBP1*. (A) *HBP1* 3′ UTR containing miR‐21 binding site was shown. (B) Western blot assay showed that the protein expression level of HBP1 in A549/PTX cells and A549/DDP cells was lower than that in parental A549 cells. (C) The relative luciferase activity in *HBP1* 3′ UTR‐wt and miR‐21 mimic cotransfection group was significantly lower than that in mimic NC group, whereas there was no significant difference in the relative luciferase activity between *HBP1* 3′ UTR‐mut and miR‐21 mimic cotransfection group and mimic NC group. *P < 0.05, compared with mimic NC group. (D) The result of western blot showed that miR‐21 mimic reduced the protein level of HBP1 in A549 cells, while miR‐21 inhibitor raised the protein expression of HBP1 in A549/PTX cells and A549/DDP cells. (E) The mechanism scheme showing miR‐21/*HBP1*/EMT signaling pathways in A549 cells was illustrated.

In five cases of A549, A549/PTX, and A549/DDP cells, expression of 8378 mRNAs was upregulated, while expression of 10,952 mRNAs was downregulated (Fig. S1D). The expression of *HBP1* in drug‐resistant A549/PTX and A549/DDP cells was significantly decreased by 3.45 times (*P *= 1.33E‐10). The heat map showed the mRNAs with the most significant differential expression (Fig. S1E). TCGA analysis displayed that patients with higher level of *HBP1* had better prognosis and longer survival time (Fig. S1F). The fact that *HBP1* was the potential target gene of miR‐21 had been authenticated through miRNA online prediction database (miRNA.org and TargetScan) (Fig. 12A). The result of western blot experiment showed that compared with parental A549 cells, the protein level of HBP1 in A549/PTX cells and A549/DDP cells was much lower (Fig. 12B). Luciferase assay showed that after the cotransfection of miR‐21 mimic and PGL3‐*HBP1*‐wt, the luciferase intensity in cells decreased (*P *< 0.05), while the cotransfection of miR‐21 mimic and PGL3‐*HBP1*‐mut did not show any significant difference from the blank group (*P *> 0.05, Fig. 12C). According to the detection of western blot, miR‐21 mimic contributed to the reduction of HBP1 protein level in A549 cells, and miR‐21 inhibitor upregulated HBP1 protein level in A549/PTX and A549/DDP cells (Fig. 12D). We have figured out how miR‐21/*HBP1*/EMT pathway influenced drug‐resistant lung adenocarcinoma cells, which is illustrated in Figure 12E that miR‐21 affected migration and invasion ability of drug‐resistant lung adenocarcinoma cells by targeting *HBP1*, therefore modulating transformation of EMT.

## Discussion

In this study, we have revealed that miR‐21 played an important part in the invasion and migration ability of drug‐sensitive or drug‐resistant lung adenocarcinoma cancer cells through targeting *HBP1*. The IC_50_ of A549/PTX cells and A549/DDP cells were obviously higher than that of A549 parental cells. Meanwhile, we also found that A549/PTX and A549/DDP cells had stronger ability of migration and invasion compared with parental A549 cells. Enforced overexpression of miR‐21 promoted the formation of EMT phenotype in parental A549 cells, whereas the knockdown of miR‐21 reversed the EMT phenotype in A549/PTX cells or A549/DDP cells. *HBP1* was an inhibitory target gene of miR‐21 and their expression levels were negatively related.

MiR‐21 level was significantly higher in nine gastric cancer cell lines than in normal gastric mucosal epithelial cell line GES‐1 27. Meanwhile, miR‐21 expression in clear cell renal cell carcinoma (ccRCC) cells was significantly higher compared with that in the HK‐2 cell line (an immortalized proximal tubule epithelial cell line from normal adult human kidney) 28. Besides, a recent study has specifically identified dysregulated miRNAs in NSCLC using next‐generation sequencing (NGS) technology, among which miR‐21 or miR‐188 overexpression correlates with a negative prognosis 29. The present study results demonstrated that miR‐21 level in PTX‐ and DDP‐resistant A549 cells was significantly higher than in A549 cells. Additionally, miR‐21 could promote the propagation and metastasis of gastric cancer cell line SGC‐7901 in vitro 27, 30. Similarly, miR‐21 upregulation increased the viability and facilitated the metastasis of HepG2 cells (hepatic cancer cell line) 31. Besides, miR‐21 promotes migration and invasion of glioma cells lines U87, A172, T98, and U343 32. In the current study, miR‐21 overexpression promoted A549 cells metastasis and miR‐21 underexpression led to the repression of cell migration and invasion.

On the other hand, miRNAs are emerging as critical core regulators of drug resistance, acting by modulating EMT and cancer‐related immune responses 33. MiR‐21 overexpression facilitates ccRCC sphere formation, in which the elevated levels of stem cell‐related transcription factors and EMT markers have been seen, and therefore promotes ccRCC tumorigenesis 28. In addition, miR‐21 inhibited TGF‐*β*1‐induced EMT in gastric cancer, leading to the progression of gastric cancer 34. The knockdown of miR‐21 repressed TGF‐*β*1‐induced fibrogenic EMT in hepatic fibrosis cell line QSG‐7701 35. It is also notable that upregulated miR‐21 by nicotine intake promoted EMT in esophageal cancer cell line EC9706, therefore inducing the aggressiveness of esophageal cancer 36. We found that the overexpression of miR‐21 promoted the formation of EMT phenotype in parental A549 cells, whereas the underexpression of miR‐21 reversed the EMT phenotype in PTX‐ or DDP‐resistant A549 cells. EMT has been recognized to be involved in acquiring drug resistance and cell aggressiveness in cancer. The knockdown of Linc‐ROR led to the decrease of EMT of docetaxel‐resistant lung adenocarcinoma cells to sensitize drug‐resistant cells to chemotherapy 37. The acquisition of pemetrexed resistance enhances EMT in vivo and in vitro 38. DDP‐resistant nasopharyngeal carcinoma cells acquired EMT characteristics, which could be responsible for the DDP resistance of nasopharyngeal carcinoma cells 39. In addition, chemoresistant NSCLC cells displayed higher mobility and more invasive capabilities as well as enhancement of EMT 40. Based on previous discoveries and our results, we thus speculated that miR‐21 might contribute to the EMT within lung adenocarcinoma cells and induce drug resistance. This has been investigated in other studies. For example, miR‐21 sustained EMT, promoted the tumor immune microenvironment formation, and conferred resistance to neoadjuvant trastuzumab and chemotherapy of HER2‐positive breast cancer cells 33. Similarly, Zhen et al. found that miR‐21 induced EMT and gemcitabine resistance in breast cancer cells 41.

*HBP1*, a tumor suppressor, has been shown to contribute to cell senescence and block tumorigenesis in several cancers 42, including lung adenocarcinoma (H1299 [p53−/−], A549), hepatocellular carcinoma (HepG2), and osteogenic sarcoma (U2OS). In addition, a previous study of Chan et al. investigated the influence of *HBP1* on invasive oral cancer 43. Suppression of *HBP1* could also promote human breast cancer cell migration and invasion 44. Taken together, alterations of the *HBP1* transcriptional repressor were associated with the invasion and migration ability of cancer cells 45, 46, which was consistent with our experiments result. The activity of *HBP1* is modulated through various mechanisms such as the activation of Wnt/*β*‐catenin signaling 47, the targeting of PI3K/FOXO pathway 48, and the regulation of abnormal gene promoter methylation 49. Furthermore, a previous study revealed that *HBP1* enhanced radiosensitivity and overcame acquired radioresistance after radiotherapy 50. In the study herein, we investigated and verified the targeted relationship between *HBP1* and miR‐21‐3p via dual luciferase report system. Based on the above studies, we may safely pose a hypothesis that miR‐21/*HBP1* axis played a significant role in invasion, migration, and transformation of EMT of drug‐resistant lung adenocarcinoma cancer cells.

## Conclusion

In conclusion, the present study demonstrated that miR‐21 was upregulated in lung adenocarcinoma cell lines with or without drug resistance. Moreover, miR‐21 promoted cell propagation, migration, invasion, and EMT transformation of A549 cells. Finally, we verified that miR‐21 could directly bind to the 3′ UTR of *HBP1*, which was found to be an important functional mediator of miR‐21 in A549 cells. Overall, our findings indicate that miR‐21 functions as a tumor facilitator in lung adenocarcinoma through targeting *HBP1* and may be a promising candidate for miR‐based therapy against lung adenocarcinoma.

## Conflict of Interest

The authors declare no potential conflicts of interest.

## Supporting information


**Figure S1.** MiRNA and mRNA expression profiling.

## References

[1] Chen, W. , R. Zheng , S. Zhang , H. Zeng , T. Zuo , C. Xia , et al. 2017 Cancer incidence and mortality in china in 2013: An analysis based on urbanization level. Chin. J. Cancer Res. 29:1–10. 28373748 10.21147/j.issn.1000-9604.2017.01.01 PMC5348470

[2] Huang, Q. , V. E. Schneeberger , N. Luetteke , C. Jin , R. Afzal , M. M. Budzevich , et al. 2016 Preclinical modeling of kif5b‐ret fusion lung adenocarcinoma. Mol. Cancer Ther. 15:2521–2529. 27496134 10.1158/1535-7163.MCT-16-0258 PMC5289739

[3] Welch, D. R. , D. J. Schissel , R. P. Howrey , and P. A. Aeed . 1989 Tumor‐elicited polymorphonuclear cells, in contrast to “normal” circulating polymorphonuclear cells, stimulate invasive and metastatic potentials of rat mammary adenocarcinoma cells. Proc. Natl Acad. Sci. USA 86:5859–5863. 2762301 10.1073/pnas.86.15.5859 PMC297730

[4] Goldstraw, P. , J. Crowley , K. Chansky , D. J. Giroux , P. A. Groome , R. Rami‐Porta , et al. 2007 The IASLC Lung Cancer Staging Project: Proposals for the revision of the TNM stage groupings in the forthcoming (seventh) edition of the TNM classification of malignant tumours. J. Thorac. Oncol. 2:706–714. 17762336 10.1097/JTO.0b013e31812f3c1a

[5] Borczuk, A. C. 2016 Prognostic considerations of the new world health organization classification of lung adenocarcinoma. Eur. Resp. Rev. 25:364–371. 10.1183/16000617.0089-2016 PMC9487552 27903658

[6] Li, H. , P. Zhang , X. Sun , Y. Sun , C. Shi , H. Liu , et al. 2015 MicroRNA‐181a regulates epithelial‐mesenchymal transition by targeting PTEN in drug‐resistant lung adenocarcinoma cells. Int. J. Oncol. 47:1379–1392. 26323677 10.3892/ijo.2015.3144

[7] Li, H. , J. He , N. Zhong , and R. M. Hoffman . 2015 Drug exposure in a metastatic human lung adenocarcinoma cell line gives rise to cells with differing adhesion, proliferation, and gene expression: Implications for cancer chemotherapy. Mol. Med. Rep. 12:3236–3242. 26004767 10.3892/mmr.2015.3837 PMC4526089

[8] Wang, Y. , C. Huang , N. R. Chintagari , D. Xi , T. Weng , and L. Liu . 2015 Mir‐124 regulates fetal pulmonary epithelial cell maturation. Am. J. Physiol. Lung Cell. Mol. Physiol. 309:L400–L413. 26071557 10.1152/ajplung.00356.2014 PMC4538233

[9] Osada, H. , and T. Takahashi . 2007 MicroRNAs in biological processes and carcinogenesis. Carcinogenesis 28:2–12. 17028302 10.1093/carcin/bgl185

[10] Lovat, F. , N. Valeri , and C. M. Croce . 2011 MicroRNAs in the pathogenesis of cancer. Semin. Oncol. 38:724–733. 22082758 10.1053/j.seminoncol.2011.08.006

[11] Hwang, J. H. , J. Voortman , E. Giovannetti , S. M. Steinberg , L. G. Leon , Y. T. Kim , et al. 2010 Identification of microRNA‐21 as a biomarker for chemoresistance and clinical outcome following adjuvant therapy in resectable pancreatic cancer. PLoS ONE 5:e10630. 20498843 10.1371/journal.pone.0010630 PMC2871055

[12] Rossi, S. , M. Shimizu , E. Barbarotto , M. S. Nicoloso , F. Dimitri , D. Sampath , et al. 2010 MicroRNA fingerprinting of CLL patients with chromosome 17p deletion identify a mir‐21 score that stratifies early survival. Blood 116:945–952. 20393129 10.1182/blood-2010-01-263889 PMC4916575

[13] Gao, W. , Y. Yu , H. Cao , H. Shen , X. Li , S. Pan , et al. 2010 Deregulated expression of mir‐21, mir‐143 and mir‐181a in non small cell lung cancer is related to clinicopathologic characteristics or patient prognosis. Biomed. Pharmacother. 64:399–408. 20363096 10.1016/j.biopha.2010.01.018

[14] Li, J. , H. Huang , L. Sun , M. Yang , C. Pan , W. Chen , et al. 2009 Mir‐21 indicates poor prognosis in tongue squamous cell carcinomas as an apoptosis inhibitor. Clin. Cancer Res. 15:3998–4008. 19509158 10.1158/1078-0432.CCR-08-3053

[15] Xie, Y. , N. W. Todd , Z. Liu , M. Zhan , H. Fang , H. Peng , et al. 2010 Altered miRNA expression in sputum for diagnosis of non‐small cell lung cancer. Lung Cancer 67:170–176. 19446359 10.1016/j.lungcan.2009.04.004 PMC2846426

[16] Li, B. , S. Ren , X. Li , Y. Wang , D. Garfield , S. Zhou , et al. 2014 Mir‐21 overexpression is associated with acquired resistance of egfr‐tki in non‐small cell lung cancer. Lung Cancer 83:146–153. 24331411 10.1016/j.lungcan.2013.11.003

[17] Berasi, S. P. , M. Xiu , A. S. Yee , and K. E. Paulson . 2004 Hbp1 repression of the p47phox gene: Cell cycle regulation via the nadph oxidase. Mol. Cell. Biol. 24:3011–3024. 15024088 10.1128/MCB.24.7.3011-3024.2004 PMC371097

[18] Zhuma, T. , R. Tyrrell , B. Sekkali , G. Skavdis , A. Saveliev , M. Tolaini , et al. 1999 Human hmg box transcription factor hbp1: A role in hcd2 lcr function. EMBO J. 18:6396–6406. 10562551 10.1093/emboj/18.22.6396 PMC1171702

[19] Pan, K. , Y. Chen , M. Roth , W. Wang , S. Wang , A. S. Yee , et al. 2013 Hbp1‐mediated transcriptional regulation of DNA methyltransferase 1 and its impact on cell senescence. Mol. Cell. Biol. 33:887–903. 23249948 10.1128/MCB.00637-12 PMC3623086

[20] Li, H. , W. Wang , X. Liu , K. E. Paulson , A. S. Yee , and X. Zhang . 2010 Transcriptional factor hbp1 targets p16(ink4a), upregulating its expression and consequently is involved in ras‐induced premature senescence. Oncogene 29:5083–5094. 20581871 10.1038/onc.2010.252

[21] Zhang, X. , J. Kim , R. Ruthazer , M. A. McDevitt , D. E. Wazer , K. E. Paulson , et al. 2006 The hbp1 transcriptional repressor participates in ras‐induced premature senescence. Mol. Cell. Biol. 26:8252–8266. 16966377 10.1128/MCB.00604-06 PMC1636767

[22] Wu, H. , R. Ng , X. Chen , C. J. Steer , and G. Song . 2016 MicroRNA‐21 is a potential link between non‐alcoholic fatty liver disease and hepatocellular carcinoma via modulation of the hbp1‐p53‐srebp1c pathway. Gut 65:1850–1860. 26282675 10.1136/gutjnl-2014-308430 PMC4882277

[23] Xu, J. , D. Liu , H. Niu , G. Zhu , Y. Xu , D. Ye , et al. 2017 Resveratrol reverses doxorubicin resistance by inhibiting epithelial‐mesenchymal transition (emt) through modulating pten/akt signaling pathway in gastric cancer. J. Exp. Clin. Cancer Res. 36:19. 28126034 10.1186/s13046-016-0487-8 PMC5270306

[24] Kitamura, K. , M. Seike , T. Okano , K. Matsuda , A. Miyanaga , H. Mizutani , et al. 2014 Mir‐134/487b/655 cluster regulates tgf‐beta‐induced epithelial‐mesenchymal transition and drug resistance to gefitinib by targeting magi2 in lung adenocarcinoma cells. Mol. Cancer Ther. 13:444–453. 24258346 10.1158/1535-7163.MCT-13-0448

[25] Rho, J. K. , Y. J. Choi , J. K. Lee , B. Y. Ryoo , S. H. Yang , C. H. Kim , et al. 2009 Epithelial to mesenchymal transition derived from repeated exposure to gefitinib determines the sensitivity to EGFR inhibitors in A549, a non‐small cell lung cancer cell line. Lung Cancer 63:219–226. 18599154 10.1016/j.lungcan.2008.05.017

[26] Bharti, R. , G. Dey , and M. Mandal . 2016 Cancer development, chemoresistance, epithelial to mesenchymal transition and stem cells: A snapshot of il‐6 mediated involvement. Cancer Lett. 375:51–61. 26945971 10.1016/j.canlet.2016.02.048

[27] Zhang, B. G. , J. F. Li , B. Q. Yu , Z. G. Zhu , B. Y. Liu , and M. Yan . 2012 MicroRNA‐21 promotes tumor proliferation and invasion in gastric cancer by targeting pten. Oncol. Rep. 27:1019–1026. 22267008 10.3892/or.2012.1645 PMC3583594

[28] Cao, J. , J. Liu , R. Xu , X. Zhu , L. Liu , and X. Zhao . 2016 MicroRNA‐21 stimulates epithelial‐to‐mesenchymal transition and tumorigenesis in clear cell renal cells. Mol. Med. Rep. 13:75–82. 26572589 10.3892/mmr.2015.4568 PMC4686059

[29] Gallach, S. , E. Jantus‐Lewintre , S. Calabuig‐Farinas , D. Montaner , S. Alonso , R. Sirera , et al. 2017 MicroRNA profiling associated with non‐small cell lung cancer: Next generation sequencing detection, experimental validation, and prognostic value. Oncotarget 8:56143–56157. 28915579 10.18632/oncotarget.18603 PMC5593550

[30] Sun, H. , P. Wang , Q. Zhang , X. He , G. Zai , X. Wang , et al. 2016 MicroRNA21 expression is associated with the clinical features of patients with gastric carcinoma and affects the proliferation, invasion and migration of gastric cancer cells by regulating noxa. Mol. Med. Rep. 13:2701–2707. 26847916 10.3892/mmr.2016.4863

[31] Mao, B. , H. Xiao , Z. Zhang , D. Wang , and G. Wang . 2015 MicroRNA21 regulates the expression of btg2 in hepg2 liver cancer cells. Mol. Med. Rep. 12:4917–4924. 26151427 10.3892/mmr.2015.4051 PMC4581755

[32] Luo, G. , W. Luo , X. Sun , J. Lin , M. Wang , Y. Zhang , et al. 2017 MicroRNA21 promotes migration and invasion of glioma cells via activation of sox2 and betacatenin signaling. Mol. Med. Rep. 15:187–193. 27909726 10.3892/mmr.2016.5971 PMC5355688

[33] De Mattos‐Arruda, L. , G. Bottai , P. G. Nuciforo , L. Di Tommaso , E. Giovannetti , V. Peg , et al. 2015 MicroRNA‐21 links epithelial‐to‐mesenchymal transition and inflammatory signals to confer resistance to neoadjuvant trastuzumab and chemotherapy in her2‐positive breast cancer patients. Oncotarget 6:37269–37280. 26452030 10.18632/oncotarget.5495 PMC4741929

[34] Li, C. , L. Song , Z. Zhang , X. X. Bai , M. F. Cui , and L. J. Ma . 2016 MicroRNA‐21 promotes tgf‐beta1‐induced epithelial‐mesenchymal transition in gastric cancer through up‐regulating pten expression. Oncotarget 7:66989–67003. 27611950 10.18632/oncotarget.11888 PMC5341852

[35] Liu, Z. , J. Wang , C. Guo , and X. Fan . 2015 MicroRNA‐21 mediates epithelial‐mesenchymal transition of human hepatocytes via pten/akt pathway. Biomed. Pharmacother. 69:24–28. 25661333 10.1016/j.biopha.2014.10.028

[36] Zhang, Y. , T. Pan , X. Zhong , and C. Cheng . 2014 Nicotine upregulates microRNA‐21 and promotes tgf‐beta‐dependent epithelial‐mesenchymal transition of esophageal cancer cells. Tumour Biol. 35:7063–7072. 24756761 10.1007/s13277-014-1968-z

[37] Pan, Y. , J. Chen , L. Tao , K. Zhang , R. Wang , X. Chu , et al. 2017 Long noncoding RNA ROR regulates chemoresistance in docetaxel‐resistant lung adenocarcinoma cells via epithelial mesenchymal transition pathway. Oncotarget 8:33144–33158. 28388536 10.18632/oncotarget.16562 PMC5464857

[38] Chiu, L. Y. , I. L. Hsin , T. Y. Yang , W. W. Sung , J. Y. Chi , J. T. Chang , et al. 2017 The erk‐zeb1 pathway mediates epithelial‐mesenchymal transition in pemetrexed resistant lung cancer cells with suppression by vinca alkaloids. Oncogene 36:242–253. 27270426 10.1038/onc.2016.195 PMC5241427

[39] Li, W. , H. Ma , J. Zhang , L. Zhu , C. Wang , and Y. Yang . 2017 Unraveling the roles of cd44/cd24 and aldh1 as cancer stem cell markers in tumorigenesis and metastasis. Sci. Rep. 7:13856. 29062075 10.1038/s41598-017-14364-2 PMC5653849

[40] An, L. , D. D. Li , H. X. Chu , Q. Zhang , C. L. Wang , Y. H. Fan , et al. 2017 Terfenadine combined with epirubicin impedes the chemo‐resistant human non‐small cell lung cancer both in vitro and in vivo through emt and notch reversal. Pharmacol. Res. 124:105–115. 28754458 10.1016/j.phrs.2017.07.021

[41] Wu, Z. H. , Z. H. Tao , J. Zhang , T. Li , C. Ni , J. Xie , et al. 2016 MiRNA‐21 induces epithelial to mesenchymal transition and gemcitabine resistance via the pten/akt pathway in breast cancer. Tumour Biol. 37:7245–7254. 26666820 10.1007/s13277-015-4604-7

[42] Chen, Y. , K. Pan , P. Wang , Z. Cao , W. Wang , S. Wang , et al. 2016 Hbp1‐mediated regulation of p21 protein through the mdm2/p53 and tcf4/ezh2 pathways and its impact on cell senescence and tumorigenesis. J. Biol. Chem. 291:12688–12705. 27129219 10.1074/jbc.M116.714147 PMC4933444

[43] Chan, C. Y. , S. Y. Huang , J. J. Sheu , M. M. Roth , I. T. Chou , C. H. Lien , et al. 2017 Transcription factor hbp1 is a direct anti‐cancer target of transcription factor foxo1 in invasive oral cancer. Oncotarget 8:14537–14548. 28099936 10.18632/oncotarget.14653 PMC5362424

[44] Li, H. , C. Bian , L. Liao , J. Li , and R. C. Zhao . 2011 Mir‐17‐5p promotes human breast cancer cell migration and invasion through suppression of hbp1. Breast Cancer Res. Treat. 126:565–575. 20505989 10.1007/s10549-010-0954-4

[45] Chen, Y. C. , X. W. Zhang , X. H. Niu , D. Q. Xin , W. P. Zhao , Y. Q. Na , et al. 2010 Macrophage migration inhibitory factor is a direct target of hbp1‐mediated transcriptional repression that is overexpressed in prostate cancer. Oncogene 29:3067–3078. 20383199 10.1038/onc.2010.97

[46] Paulson, K. E. , K. Rieger‐Christ , M. A. McDevitt , C. Kuperwasser , J. Kim , V. E. Unanue , et al. 2007 Alterations of the hbp1 transcriptional repressor are associated with invasive breast cancer. Can. Res. 67:6136–6145. 10.1158/0008-5472.CAN-07-0567 17616670

[47] Wan, Y. C. , T. Li , Y. D. Han , H. Y. Zhang , H. Lin , and B. Zhang . 2016 MicroRNA‐155 enhances the activation of wnt/beta‐catenin signaling in colorectal carcinoma by suppressing hmg‐box transcription factor 1. Mol. Med. Rep. 13:2221–2228. 26780942 10.3892/mmr.2016.4788

[48] Coomans de Brachene, A. , E. Bollaert , A. Eijkelenboom , A. de Rocca Serra , K. E. van der Vos , B. M. Burgering , et al. 2014 The expression of the tumour suppressor hbp1 is down‐regulated by growth factors via the pi3k/pkb/foxo pathway. Biochem. J. 460:25–34. 24762137 10.1042/BJ20131467

[49] Tseng, R. C. , W. R. Huang , S. F. Lin , P. C. Wu , H. S. Hsu , and Y. C. Wang . 2014 Hbp1 promoter methylation augments the oncogenic beta‐catenin to correlate with prognosis in nsclc. J. Cell Mol. Med. 18:1752–1761. 24895061 10.1111/jcmm.12318 PMC4196651

[50] Chen, Y. , Y. Wang , Y. Yu , L. Xu , Y. Zhang , S. Yu , et al. 2016 Transcription factor hbp1 enhances radiosensitivity by inducing apoptosis in prostate cancer cell lines. Anal. Cell. Pathol. 2016:7015659. 10.1155/2016/7015659 PMC4749775 26942107

