# Supplementary figures and images for: MiR‐21 improves invasion and migration of drug‐resistant lung adenocarcinoma cancer cell and transformation of EMT through targeting HBP1
Source: Cancer Med. 2018 Apr 16;7(6):2485–503. doi: 10.1002/cam4.1294 (PMC6010699; doi:10.1002/cam4.1294)

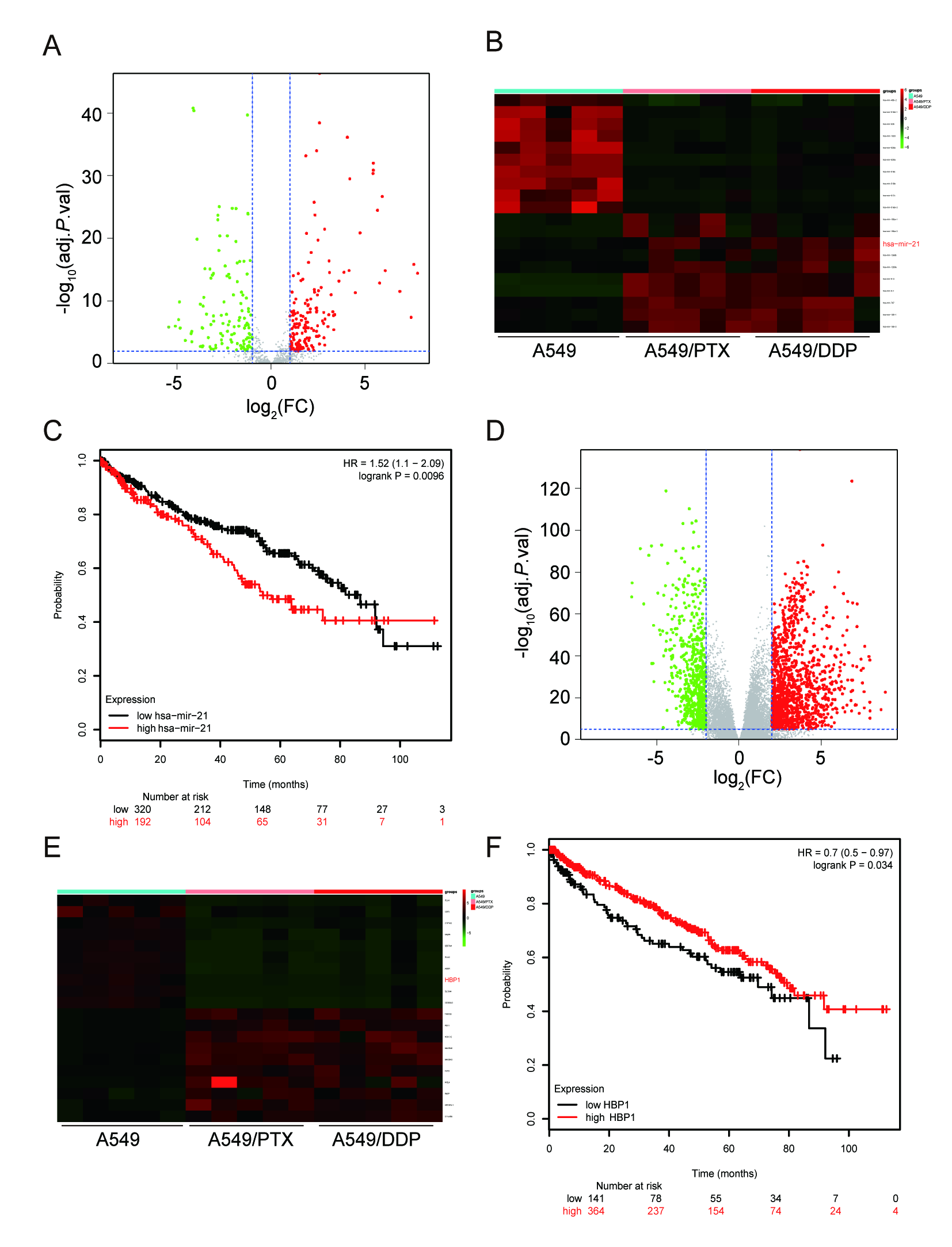

Supplement: Supplementary file 1 — Figure S1. MiRNA and mRNA expression profiling. [file CAM4-7-2485-s001.tif]
